# Low-glucose enhances keratocyte-characteristic phenotype from corneal stromal cells in serum-free conditions

**DOI:** 10.1038/srep10839

**Published:** 2015-06-03

**Authors:** James W. Foster, Ricardo M. Gouveia, Che J. Connon

**Affiliations:** 1 Johns Hopkins School of Medicine, Baltimore, MD, USA; 2 Institute of Genetic Medicine, Newcastle University, Newcastle-upon-Tyne, UK

## Abstract

The avascular cornea is a uniquely-isolated organ, with its stroma constituting a nutrient-poor environment. Consequently, the availability of metabolites such as glucose to corneal stromal cells is considerably reduced compared with other tissues, or indeed with media commonly used to culture these cells *in vitro*. However, the role of glucose in the behaviour of human corneal keratocytes has been overlooked. As such, we sought to investigate the effects of low-glucose formulations on the phenotype of human corneal stromal cells. Cells cultured in low-glucose were able to survive for extended periods when compared to high-glucose, serum-free conditions. Furthermore, low-glucose enhanced their reversal to a keratocyte-characteristic phenotype. Specifically, cells within low-glucose medium assumed dendritic morphologies, with bean-shaped condensed nuclei, absence of alpha-smooth muscle actin or stress fibres, and a corresponding reduction in migratory and contractile activities when compared with high-glucose, serum-free conditions. Moreover, cells within low-glucose uniquely recovered the ability to express a robust keratocyte-characteristic marker, CD34, while still expressing elevated levels of other representative phenotypic markers such as keratocan, lumican, ALDH1A1, and ALDH3A1. These results indicate that low-glucose enhances keratocyte-characteristic phenotype above and beyond established media formulations and thus has important implications for corneal biology in health and disease.

The transparency of the cornea is contingent on the preservation of the light-refracting corneal stroma, a tissue largely comprised by keratocytes embedded within a highly-ordered array of extracellular matrix (ECM) proteins. As such, the corneal stroma is effectively an isolated tissue within the body, devoid of blood supply and compartmentalized between the endothelial and the stratified epithelial cells^1^. Keratocytes at the centre of the corneal stroma can be over 5 mm away from any blood supply and > 400 μm from endothelial support^1^. Due to these physical constraints keratocytes exist in a persistently nutrient-poor environment between the collagen lamellae of the corneal stroma^2^. The majority of nutrients reaching resident keratocytes come from the aqueous humour, where they are selectively transported by endothelial cells into the stroma proper^3^,^4^. The majority of the stromal keratocytes are located in the anterior third of the stroma where damage, requiring repair, is most likely. However, this localization is theoretically where availability of glucose and other metabolites is most limited.

Strikingly, the 4.5 g.L^−1^ of glucose in commercially-available media formulations such as Dulbecco’s modified eagle’s medium (DMEM) corresponds to a 5 to 9-fold higher concentration compared to that in the human cornea (previously estimated to vary between 0.9-0.5 g.L^−1^)^2^,^5^. Glucose is not only an important nutrient and source of energy for the cells but also a fundamental regulator of cell phenotype *in vivo* and *in vitro*, acting through multiple signalling cascades^6^,^7^,^8^. It is perhaps unsurprising, therefore, that keratocytes cultured *in vitro* under typical media formulations (i.e., with relatively high glucose concentrations) fail to maintain the phenotype they characteristically present in the unwounded cornea or even immediately after isolation. An excess of metabolites is known to affect the expression of ECM proteins in other cell types both *in vivo* and *in vitro*^9^,^10^,^11^. For keratocytes in particular, the expression of characteristic small leucine-rich proteoglycans (SLRPs) such as keratocan and lumican, and of corneal crystallins such as aldehyde dehydrogenases 1A1 and 3A1 (ALDH1A1 and ALDH3A1, respectively) is rapidly lost upon exposure to standard tissue culture conditions^12^,^13^,^14^. The resulting cells also lose their keratocyte-characteristic dendritic morphology and become more fibroblastic in nature, with a corresponding increased expression of alpha-smooth muscle actin (αSMA)^15^. This keratocyte-fibroblast transition has traditionally been attributed to the presence of serum products within the culture media, a notion supported by several studies where the use of serum-free media induced a marked reversal of cell phenotype^16^,^17^,^18^,^19^. This reversal is usually characterized by increased expression of keratocyte markers, namely keratocan^14^,^19^, alongside the absence of the myofibroblastic marker αSMA^17^,^20^.

In this context, the present study aims to understand the effects of glucose on corneal stromal cells cultured *in vitro* and corresponding reversal to a keratocyte-characteristic phenotype, through the analysis of cell morphology, proliferation, migration, contractibility, and expression of specific keratocyte markers. This work addresses the theory that, by recapitulating the nutrient-poor environment of the normal corneal stroma *in vitro* (i.e., using low-glucose and serum-free conditions), it is possible to maintain corneal stromal cells with a phenotype closer to that observed in the native, undamaged tissue. Such control has multiple applications in fundamental, biotechnological, and clinical research, specifically in the investigation of corneal biology in health and disease states, and for the production of corneal bio-prosthetic equivalents.

## Results

### Low-glucose promotes dendritic morphology and survival of human corneal stromal cells in serum-free conditions

**Figure 1:**
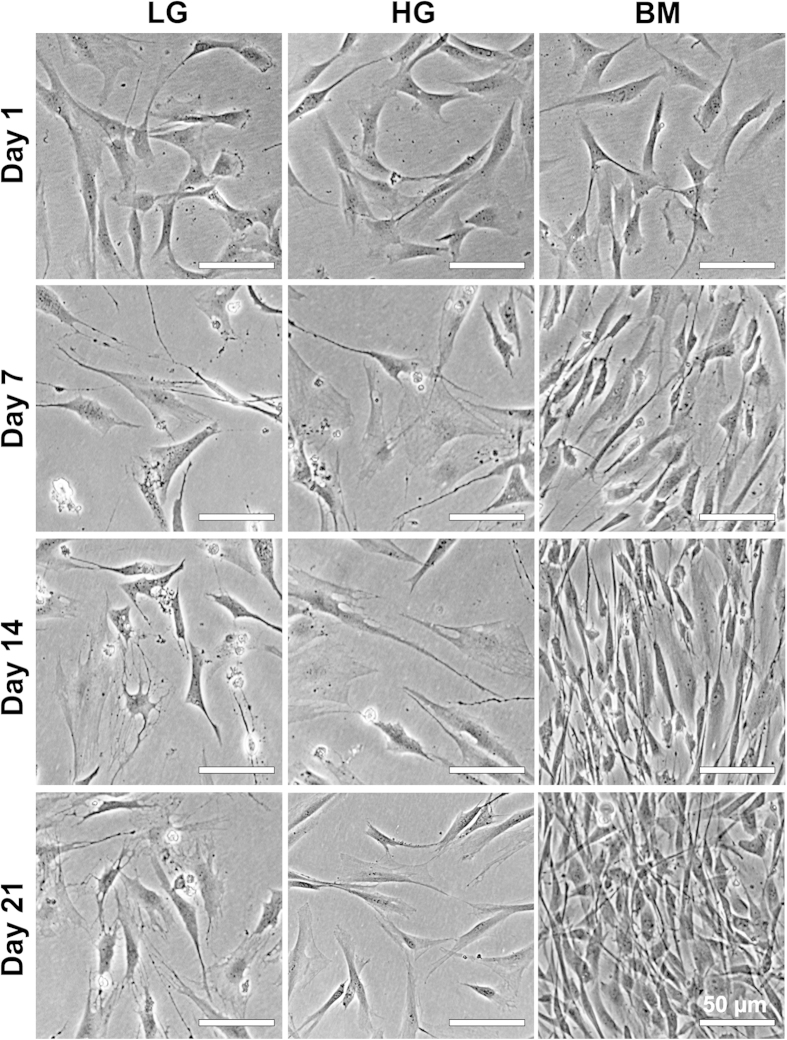
Effects of glucose concentration on human corneal stromal cell morphology. Human corneal stromal cells were grown in serum-free media containing glucose at 2 (LG) or 4.5 g.L^−1^ (HG), or in serum-containing medium (BM) for up to 21 days. Panels show representative images of three independent experiments. Scale bars = 50 μm.

**Figure 2:**
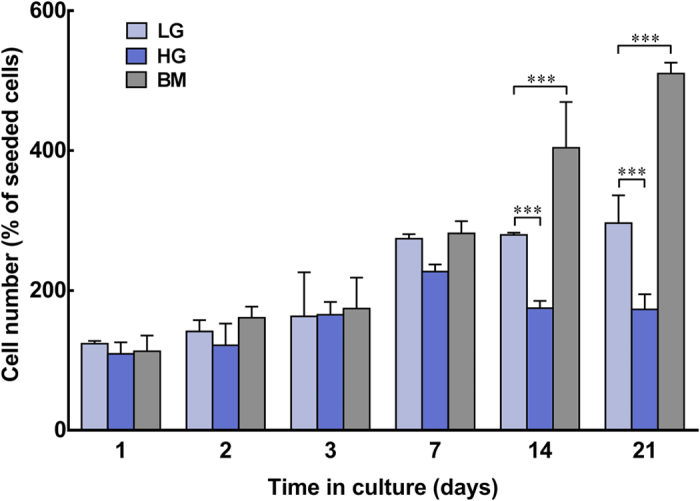
Effects of glucose concentration on human corneal stromal cell proliferation. Human corneal stromal cells were grown in serum-free media containing glucose at 2 (LG; *light blue*) or 4.5 g.L^−1^ (HG; *dark blue*), or in serum-containing medium (BM; *grey bars*) for up to 21 days. Total number of live cells was determined using the Alamar blue assay, normalised against the number of cells initially seeded, and expressed as average ± S.D. of three independent experiments (*n* = 3); *** corresponded to *p* < 0.001.

**Figure 3:**
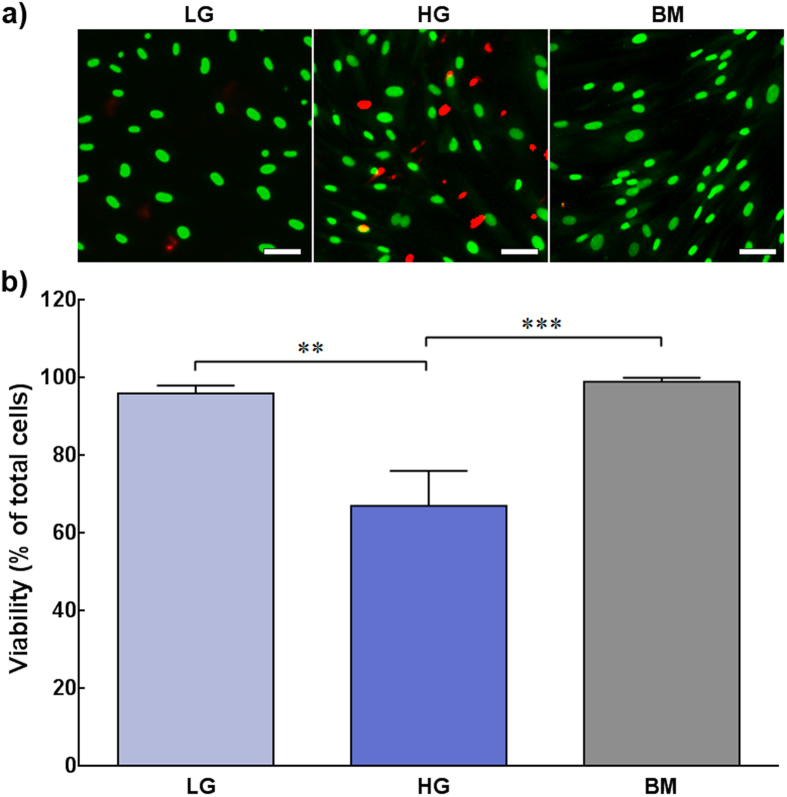
Effects of glucose concentration on human corneal stromal cell viability. Human corneal stromal cells grown for 21 days in serum-free media containing glucose at 2 (LG) or 4.5 g.L^−1^ (HG), or in serum-containing medium (BM) were analysed using the live/dead double staining assay and imaged by fluorescence microscopy. **a**) Representative images of cultures maintained in LG, HG, and BM conditions, evidencing live (*green*) and dead cells (red). **b**) Cell viability calculated as percentage of live cells in LG (*light* blue), HG (*dark blue*), and BM (*grey*) cultures. Data was expressed as average ± S.D. of three independent experiments (*n* = 3); ** and *** corresponded to *p* < 0.01 and 0.001, respectively.

To begin exploring the effects of glucose on the phenotype of human corneal stromal cells, cultures were maintained for 21 days in low- or high-glucose serum-free media (LG or HG, with corresponding 2 or 4.5 g.L^−1^ of *D*-glucose, respectively), or in basal medium (BM, containing 5% FBS), and analysed for cell proliferation, viability, and morphology. The morphology of cells cultured in LG, HG, and BM showed pronounced differences (Fig. 1). Phase-contrast micrographs showed that cells in serum-free conditions acquired a flattened, dendritic-shaped morphology. In LG conditions, this became the prevalent phenotype after 14 days in culture (Fig. 1). Dendritic-shaped cells were also observed in HG conditions at day 7 and 14, but almost absent at day 21 (Fig. 1). In contrast, cells grown in FBS-containing BM presented a fusiform shape characteristic of fibroblasts, and upon achieving confluence at day 7 started to stratify (Fig. 1, Day 14 and 21). In terms of proliferation, LG conditions at day 7 showed a 2.7-fold increase in the number of live cells compared to initial seeding density, a value kept constant for the remaining period of culture (Fig. 2, *light blue bars*). In contrast, cells in BM conditions continued to proliferate even after reaching confluence at day 7. At day 14 and 21, the number of live cells in BM cultures was 1.4 and 1.7-fold higher than that of LG cultures at corresponding time points (Fig. 2, *grey bars*; *p* = 0.009 and 0.0008, respectively). However, cells in HG not only stopped proliferating at day 7, but also showed a significant decrease of their numbers at later periods of culture, with the number of viable cells at day 14 and 21 reduced to approximately 60% of that from LG conditions (Fig. 2, *blue bars*; *p* = 0.0005 and 0.0006, respectively). This decrease might be attributed to impaired cell survival. Cultures analysed by the live/dead double staining assay at day 21 showed that only 67 ± 7% of cells in HG conditions were alive. This value was significantly lower in LG or BM conditions, which correspondingly maintained 96 ± 3 and 99 ± 1% of live cells (Fig. 3; *p = *0.0017 and 0.0009, respectively). However, cells cultured with 1 g.L^−1^ of glucose showed impaired proliferation and viability after only seven days in culture (Supplementary Fig. S1). Taken together, these results showed that human stromal cells cultured in serum-free conditions acquired a non-proliferative dendritic phenotype characteristic of keratocytes, but only in LG were these cells able to survive for extended periods in culture. Moreover, after 21 days in HG, cells were mainly non-dendritic, suggesting that dendritic cells were less prone to survive in these conditions.

### Low-glucose enhances the expression of keratocyte-characteristic molecular markers

**Figure 4:**
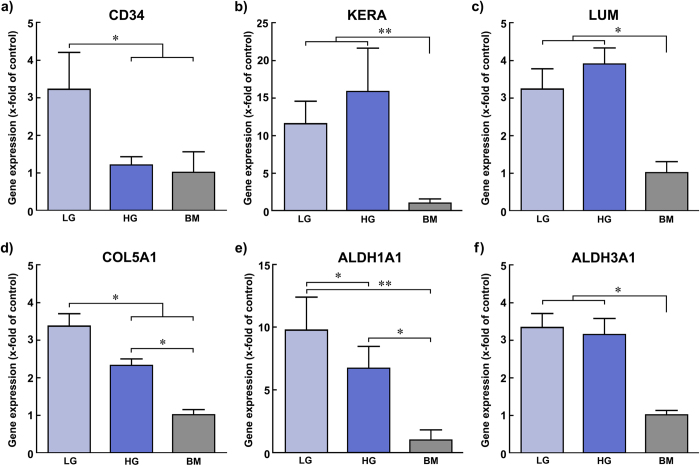
Effects of glucose concentration on the expression of keratocyte-characteristic gene markers. Human corneal stromal cells grown in serum-free media containing glucose at 2 (LG; *light blue*) or 4.5 g.L^−1^ (HG; *dark blue*), or in serum^-^containing medium (BM; *grey*) were analysed by quantitative PCR (qPCR). Relative transcription levels of genes coding for the keratocyte-characteristic markers **a**) CD34 (*CD34*), **b**) keratocan (*KERA*), **c**) lumican (*LUM*), **d**) collagen type-V (*COL5A1*), **e**) aldehyde dehydrogenase 1 A1 (*ALDH1A1*), and f) aldehyde dehydrogenase 3 A1 (*ALDH3A1*) were evaluated after normalisation against transcription of *POLR2B* housekeeping gene and primer efficiency. Values represent average ± S.D. of five independent experiments (*n* = 5). Statistical analysis was performed for comparison between serum-free vs. serum-containing media (i.e., LG or HG vs. BM) and low-glucose vs. high-glucose conditions (i.e., LG vs. HG); * and ** corresponded to *p* < 0.05 and 0.01, respectively.

**Figure 5:**
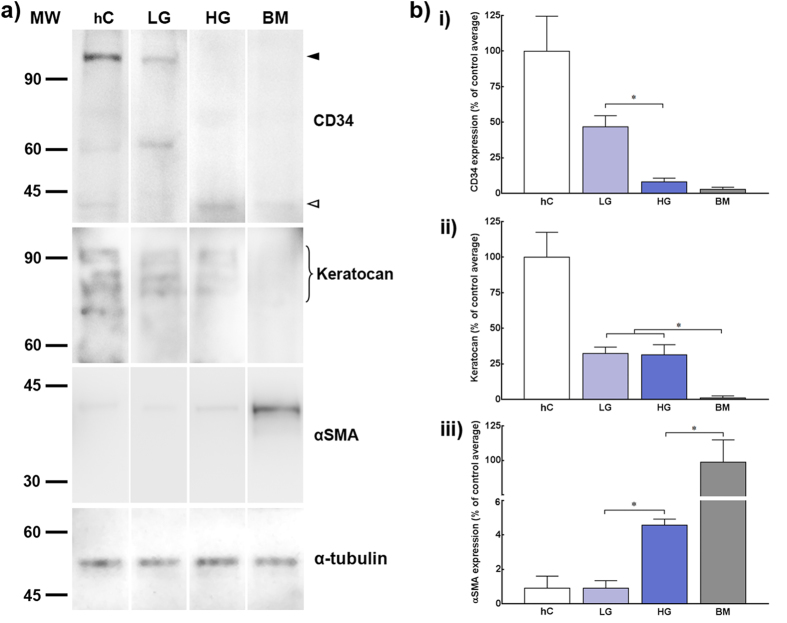
Effects of glucose concentration on the expression of keratocyte-characteristic protein markers. **a**) The expression levels of CD34, keratocan, and αSMA protein markers was analysed from lysates obtained from human corneal tissue (hC) and human corneal stromal cells grown in LG, HG, or serum-containing BM by immunoblotting. Detection of α-tubulin was performed to exclude variations from total protein load. Migration was evaluated using pre-stained molecular ladder (MW) to distinguish between high- (*white*) and low-migratory protein forms (*black arrowhead*). Panels show representative images from three independent experiments. **b**) The expression of CD34 (i), keratocan (ii), and αSMA (iii) was quantified by densitometry, normalized with α-tubulin signal, and compared between LG (*light blue*) and HG (*dark blue bars*), with expression from human corneal tissue (hC, *white*) and cells grown in BM (*grey bars*) used as reference. Data was expressed as average ± S.D. of three independent experiments (*n* = 3); * corresponded to *p* < 0.05.

**Figure 6:**
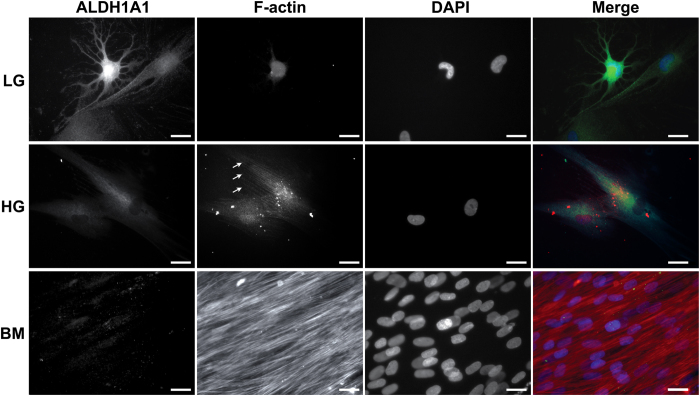
Fluorescence microscopy analysis of human corneal stromal cells grown in LG, HG, or BM. Human corneal stromal cells grown in LG, HG, or BM were analysed for ALDH1A1 (*green*), F-actin (*red*), and DAPI-stained nuclei (*blue*) by fluorescence microscopy. Cells maintained in LG were mostly dendritic, with diffuse actin signal and bean-shaped nuclei. Cells in HG showed faint ALDH1A1 cytoplasmic staining that closely localized with F-actin fibres (*arrows*). Cell grown in BM showed little ALDH1A1 signal but very evident F-actin stress fibres. Panels show representative images of five independent experiments. Scale bars = 20 μm.

To further test the effects of glucose concentration in serum-free media, the molecular phenotype of human stromal cells grown in LG, HG, and BM was analysed at both the transcriptional and protein levels. In particular, the relative expression levels of genes coding for keratocyte-characteristic markers such as CD34 *(CD34)*, keratocan (*KERA*) and lumican (*LUM*), pro-alpha 1 collagen V (*COL5A1*), and aldehyde dehydrogenases 1A1 (*ALDH1A1*) and 3A1 (*ALDH3A1*) were evaluated by qPCR (Fig. 4). The transcription of *CD34*, a gene coding for a glycoprotein posited as an alternate marker of keratocytes that is lost during *in vitro* culture of corneal stromal cells^21^, was up-regulated solely in LG cells, showing a significant 3.3 ± 0.9-fold increase in expression compared to HG or BM conditions (Fig. 4a; *p* = 0.024). This difference was also observed at the protein level (Fig. 5a), with LG inducing a significant up-regulation in CD34 that corresponded to a 5.8 ± 1.0-fold increase expression compared to HG conditions (Fig. 5b, *p* = 0.0391). Importantly, CD34 from both corneal tissue and LG lysates migrated as a protein of approximately 110 kDa (Fig. 5a, *black arrowhead*), but of only 40 kDa in HG and BM (Fig 5a, *white arrowhead*). For the remaining markers analysed, cells grown in the absence of serum (LG and HG media) showed significant up-regulation compared to the serum-containing control, BM (Fig. 4b–f). In particular, the expression of keratocan at both transcriptional (Fig. 4b) and protein levels (Fig. 5) was shown to be similar between LG and HG (Fig. 4b,5b; *p* = 0.191 and 0.34, respectively). Similarly, the expression of *LUM* and *ALDH3A1* was significantly increased in serum-free conditions, but not altered due to glucose concentrations (Fig. 4c,f). However, LG significantly enhanced transcription of *COL5A1* and *ALDH1A1* by approximately 1.4 ± 0.1 and 1.5 ± 0.4-fold compared to HG conditions (Fig. 4d,e; *p* = 0.0442 and 0.0466, respectively). Moreover, the immunofluorescence microscopy analysis of cells grown in LG showed differences in ALDH1A1 protein abundance and localisation compared to HG (Fig. 6). In particular, most cells in LG conditions were strongly ALDH1A1-positive, with bean-shaped condensed nuclei, and diffuse actin staining (Fig. 6, *upper panel*). In contrast, cells maintained in HG presented a fainter ALDH1A1 staining that closely localised with actin stress fibres (Fig. 6, *middle panel*). The actin stress fibres observed in HG were similar to those observed for cells in BM (Fig. 6, *lower panel*), albeit less pronounced.

Furthermore, the expression of alpha smooth muscle actin (αSMA), a robust marker for keratocyte activation into the myofibroblastic contractile phenotype^15^,^22^ was analysed at the protein level for all culture conditions (Fig. 5). Previously, it was shown that αSMA expression is up-regulated in corneal stromal fibroblasts by the presence of serum in the culture media, but minimal in serum-free conditions^20^. As expected, the expression of αSMA from corneal stromal cells grown in BM was significantly higher compared to that from cells maintained in serum-free conditions (Fig. 5; *p* = 0.0001). However, αSMA expression was also affected by glucose concentration, with cells maintained in LG showing normalised αSMA levels comparable to those of corneal tissue extracts, which corresponded to a significant 4-fold reduction compared to HG conditions (Fig. 5b, *p* = 0.0422). Taken together, these results showed that LG allowed, and in some cases enhanced, the serum-free-induced expression of keratocyte-characteristic markers, whilst eliminating the formation of cytoskeleton elements associated with keratocyte activation, migration, and contraction.

### Low-glucose inhibits corneal stromal cell migration and contraction

**Figure 7:**
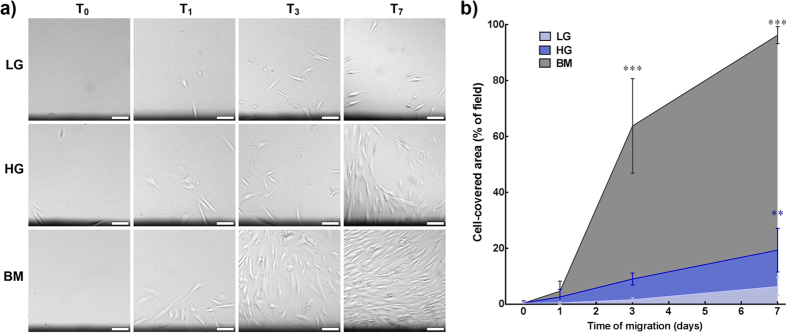
Effect of glucose concentration on human corneal stromal cell migration. Serum-deprived human corneal stromal cells were seeded onto the bottom-half of wells from polystyrene culture plates and maintained in LG, HG, or BM for 7 days. **a**) Representative images of cell migration at the start and day 1, 3, and 7 of the experiment (T_0-7_, respectively). Cell migration was assessed by time-lapse bright-field microscopy by imaging the movement of cells from their original seeding site (i.e., corresponding to the shadowed areas at the bottom of the micrographs) towards the adjacent cell-free area. Scale bars = 100 μm. **b**) Migration quantified as the percentage of initially cell-free surface covered by cells in LG (*light blue*), HG, (*dark blue*), and BM (*grey area*) at different time points. Data was expressed as average ± S.D. of three replicates from three independent experiments (*n* = 3); ** and *** corresponded to *p* < 0.01 and 0.001, respectively.

The effects of LG on the expression of f-actin and αSMA motivated further analysis of the impact of glucose concentration on corneal stromal cell migration and contraction. As such, serum-deprived cells were seeded onto the lower-half surface of polystyrene wells tilted to 45° at below-confluence levels, and then maintained in LG, HG, or BM for 7 days and monitored throughout by phase-contrast microscopy (Fig. 7). The results showed that cell migration was reduced in LG compared to HG conditions (Fig. 7a). At day 7, cells in LG occupied 7.2 ± 3.8% of the previously cell-free surface, a significantly lower coverage compared to that in HG or BM conditions (Fig. 7b; *p* = 0.0017 and 0.0001, respectively), covering 21.0 ± 9.5 and 95.7 ± 3.1% of the corresponding initial cell-free area.

**Figure 8:**
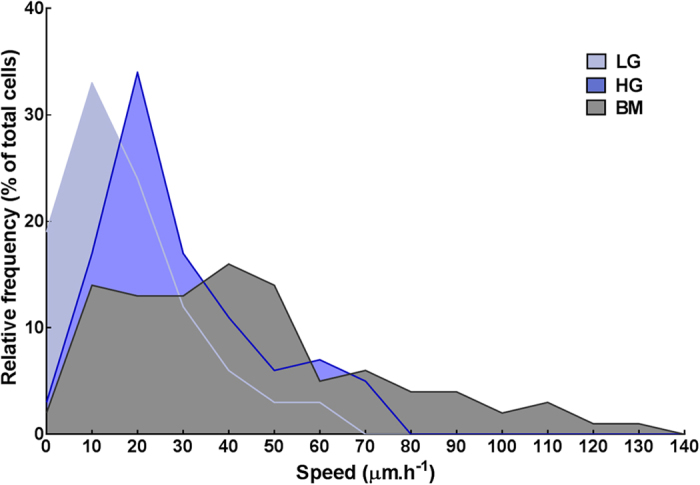
Effect of glucose concentration on the speed of human corneal stromal cells. Serum-deprived human corneal stromal cells were used to evaluate migration were imaged by time-lapse bright-field microscopy. The absolute cell speed (μm.h^−1^) was evaluated from tracing the movement of 100 cells in each condition, using micrographs taken in 5-min intervals during the initial 24 h in culture. Data was grouped in 10 μm.h^−1^ speed intervals, and expressed as the average from three independent experiments using cells from independent donors (*n* = 3).

In addition, to obviate the contribution of cell proliferation in the occupancy of cell-free surfaces, the movement of cells was tracked and quantified by time-lapse microscopy in the period immediately after seeding (Fig. 8). The results showed that LG, HG, and BM cultures have a different speed distribution pattern, with cells maintained in low-glucose conditions showing significantly slower average speeds (17.7 ± 14.0 μm.h^−1^) compared to cells in HG (28.7 ± 17.5 μm.h^−1^; *p* = 0.0029) or BM conditions (45.8 ± 33.1 μm.h^−1^; *p* = 0.0001).

**Figure 9:**
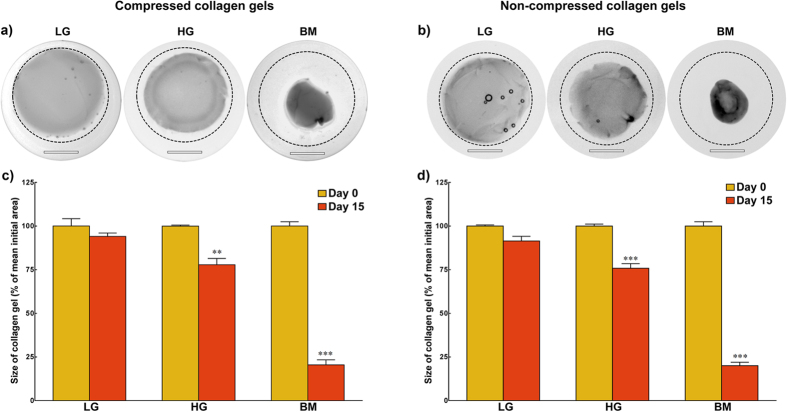
Effect of glucose concentration on the contractile activity of human corneal stromal cells. **a**) Compressed and **b**) non-compressed collagen gels embedding serum-deprived human corneal stromal cells were imaged after incubation in LG (*left*), HG (*centre*), and BM (*right panels*) for 15 days. Contraction was evidenced by superposition of corresponding initial size of gels (*traced line*). Scale bars = 1 cm. Cell contractile activity was evaluated by the contraction of **c**) compressed and **d**) non-compressed gels, quantified as the variation in collagen gel size before (*yellow*) and after 15 days in culture (*red bars*). Data was expressed as average ± S.D. of duplicates from three independent experiments (*n* = 3); ** and *** corresponded to *p* < 0.01 and 0.001, respectively.

Furthermore, the impact of glucose concentration on the contractile behaviour of corneal stromal cells was determined using collagen gels as a cell-encapsulating matrix (Fig. 9). Serum-deprived corneal stromal cells were embedded within compressed and non-compressed collagen gels and then maintained in LG, HG, or BM to potentiate gel contraction (Fig. 8a,b, respectively). As negative controls for contraction, gels without embedded cells were maintained in corresponding media conditions. The results showed that compressed gels cultured in LG for 15 days maintained 95 ± 2% of their original size, whereas cells in HG and BM induced gels to contract to 74 ± 4 and 20 ± 3% of their original size, respectively (Fig. 9c, *red bars*). Similar effects were observed in non-compressed collagen gels (Fig. 9b), with cells maintained in LG, HG, and BM inducing reduction of gel size to 92 ± 3, 76 ± 3, and 20 ± 2 of initial area, respectively (Fig. 9d). Gel contraction in HG and, to a greater extent, in BM was deemed significant (Fig. 9c, *p* = 0.0091 and 0.0002; Fig. 9d, *p* = 0.001 and 0.0002, respectively) and mainly derived from cell contractile activity, as their cell-free counterparts showed no variation in size (0 ± 3 and 1 ± 4% contraction in HG and BM, respectively) (Supplementary Fig. S2). In contrast, contraction in LG was not deemed significantly different between cell-embedded compressed and non-compressed gels and their cell-free counterparts (*p* = 0.0944 and 0.072, respectively), the latter showing at day 15 a 0 ± 4% variation relative to their original size (Supplementary Fig. S2).

### Low-glucose effects can be reproduced by increasing cAMP levels in high-glucose conditions

**Figure 10:**
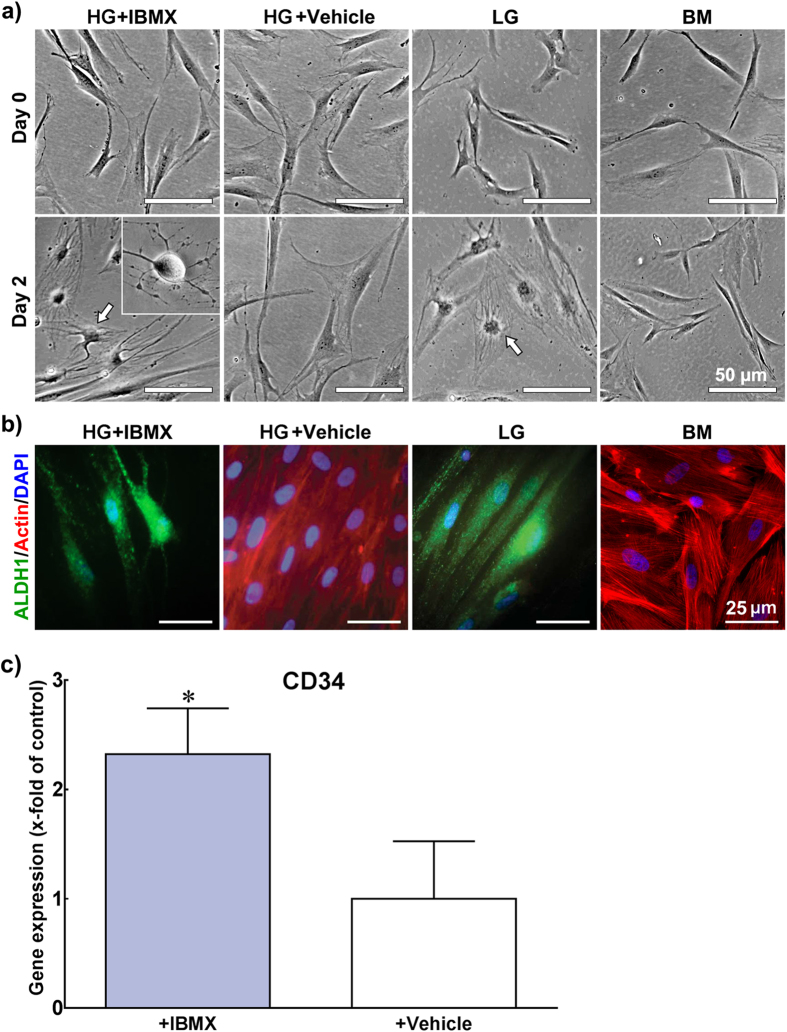
Emulation of LG conditions through increased cAMP levels in IBMX-supplemented HG medium. **a**) Human corneal stromal cells grown for 2 days in IBMX- or DMSO-supplemented HG (HG + IBMX or HG + vehicle, respectively) were analysed by phase-contrast microscopy and compared to those maintained in LG or BM conditions. Representative micrographs of IBMX-treated cells presented dendritic-type morphologies (*inset*) comparable to those in LG (*arrows*), whereas cells in HG + vehicle and BM control conditions retained their fibroblastic shape. Scale bars = 50 μm. **b**) Representative fluorescence micrographs of three independent HG + IBMX, HG + vehicle, LG, and BM cultures. The expression and localisation of ALDH1A1 (*green*) and F-actin (*red*) from cells in HG + IBMX conditions was comparable to that in LG after 2 days in culture, and at later culture stages (Fig. 6), but different from HG + vehicle cells. Cell nucleus was stained with DAPI (*blue*). Scale bars = 25 μm. **c**) IBMX-treated cells analysed by qPCR showed enhanced transcription of *CD34* compared to that of + vehicle controls. Data represents average ± S.D. of three independent experiments (*n* = 3) after normalisation with *POLR2B* housekeeping gene transcription and primer efficiency; * corresponded to *p* < 0.05.

To further understand the molecular basis of the effects induced by LG, the role of glucose-regulated signalling pathways was explored. All promoter regions of the genes tested as markers in this study were shown to contain elements responsive to cyclic adenosine monophosphate (cAMP), a potent second messenger shown to be regulated by glucose^8^. In addition, recent studies have shown that increased cAMP levels inhibited TGFβ1-induced corneal keratocyte-myofibroblastic transformation^23^ and fibrosis^24^. As such, 3-isobutyl-1-methylxanthine (IBMX), a competitive non-selective inhibitor of phosphodiesterase that raises intracellular cAMP levels^25^ was added to HG medium at 5 × 10^−6^ M (HG + IBMX), and tested for its ability to emulate the effects of LG. The morphology and molecular phenotype of human corneal stromal cells grown in HG + IBMX or DMSO vehicle control (HG + vehicle) conditions were compared to those in LG or BM (Fig. 10). Cells cultured in HG + IBMX assumed dendritic-type morphologies (Fig. 10a, *inset*) comparable to those observed in LG conditions as early as after 2 days in culture (Fig. 10a, *arrows*). In contrast, at the same time point, cells maintained in HG + vehicle or BM controls retained their fibroblast-characteristic shape (Fig. 10a). In addition, these rapid morphological changes were accompanied by alterations in ALDH1A1 protein expression and localisation relative to HG + vehicle controls (Fig. 10b), and comparable to those observed in LG conditions at later culture stages (Fig.6, *merge*). Furthermore, IBMX induced a significant increase in *CD34* transcription (Fig. 10c; *p* = 0.0192). However, in the conditions tested, IBMX did not significantly alter the transcription levels of other markers expected to be directly regulated by cAMP (i.e., keratocan; data not shown). Overall, these results showed that elevated cAMP levels induced by IBMX in a HG background were capable of reproducing some, but not all the effects induced by LG medium alone.

### Low-glucose allows scratch-wound recovery in serum-free media conditions

**Figure 11:**
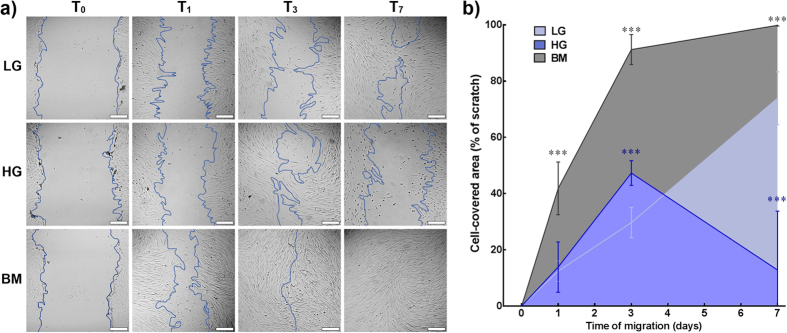
Effect of glucose concentration on scratch-wound closure. Serum-deprived human corneal stromal cells seeded as a monolayer were scratched and then maintained in LG, HG, or BM for 7 days. **a**) Representative phase-contrast micrographs of scratch-wound immediately after injury (T_0_) and 1, 3, and 7 d**a**ys afterwards (T_1–7_, respectively). Wound borders were delimited by the blue lines. Scale bars = 200 μm. **b**) Scratch-wound closure was quantified as the percentage of initial scratch surface area covered by cells in LG (*light blue*), HG, (*dark blue*), and BM (*grey area*) at different time points. Data was expressed as average ± S.D. of three replicates from three independent experiments (*n* = 3); *** corresponded to *p* < 0.001.

The possible implications of LG conditions in corneal wound healing were investigated using a scratch-wound functional assay. For that, serum-deprived corneal stromal cells in monolayer were scratched and then incubated in LG, HG, and BM for 7 days (Fig. 11). Cells started to migrate towards the cell-free area immediately after the scratch (Fig. 11a, T_1_), with LG, HG, and BM conditions showing 30 ± 5, 48 ± 5, and 91 ± 5% coverage 3 days after injury, respectively. At this time point, cells in LG showed significantly slower coverage compared to HG or BM conditions (Fig. 11b; *p* = 0.0098 and 0.0001, respectively). Afterwards, cells in the presence of serum were able to completely close the wound at day 7, whereas cells in LG conditions continued to migrate and covered 75 ± 9% of the initial area (Fig. 11a). However, in HG conditions at day 7, cells within or in close proximity of the scratch stopped migrating, became rounded, and detached, accounting for a decrease in coverage area to 13 ± 22% (Fig. 11a,b). Interestingly, this effect was circumscribed to the immediate vicinity of the initial injury, with cells from other regions of the monolayer maintaining high viability (Supplementary Fig. S3). Together with the observations obtained from long-term cultures (Fig. 1, 2, 3), these results indicated that, in serum-free conditions, corneal stromal cells are less prone to survive stress events when cultured in a HG environment. Inversely, LG conditions showed that corneal stromal cells can be maintained for more extended periods in culture, even after being challenged by injury.

## Discussion

This study sought to test the effects of low-glucose concentrations on the phenotype of stromal cells isolated from human corneal tissue and grown *in vitro*. The rationale behind these experiments was based on the premise that native corneal keratocytes occur in a nutrient-poor environment *in vivo*, where the concentration of glucose is much lower than that of commonly-used serum-free media formulations^2^. Previously, it was demonstrated that the absence of serum in the medium elicited a partial recovery of the native keratocyte phenotype from corneal fibroblasts expanded *in vitro*. This recovery increased expression of keratocyte-characteristic markers such as the SLRPs keratocan and lumican^14^,^26^, collagen type-V^19^, and crystallins ALDH1A1 and 3A1^27^,^28^,^29^ whilst inhibiting the expression of myofibroblastic αSMA^15^. However, the absence of serum in the medium was also shown to impair long-term cultures of these cells due to decreased cell adhesion and viability^30^,^31^.

The present report has now shown that, down to a point, reduced levels of glucose in serum-free conditions allowed human corneal stromal cells to survive for prolonged periods in culture without compromising cell viability. Moreover, cells maintained in LG conditions showed a further and significant increased expression of keractocyte-characteristic markers and reduced expression of stress fiber-forming αSMA. Concurrently, LG conditions reduced the migratory and contractile activity of these cells compared to HG medium, without impairing their capacity to endure and respond in an injury model. Interestingly, and despite closer to the concentrations reported for the corneal stroma^2^,^5^, glucose at 1 g.L^−1^ was not sufficient to sustain viable corneal stromal cell cultures. This might be due to the differences in glucose uptake and/or utilization between quiescent and proliferative insulin-sensitive cells^32^, or between *in vivo* and *in vitro* conditions^33^.

Prominently, glucose was shown to have an important role in corneal stromal cell morphology and survival. Despite the absence of serum in the medium, cells cultured long-term in low-glucose conditions kept high viability levels while maintaining a dendritic phenotype, with bean-shaped condensed nuclei and diffuse F-actin distribution, similar to that seen in keratocytes in the native tissue^15^. The reduced cell viability shown by these cells in HG conditions could be due to a greater susceptibility to high-energy metabolic states. Considering that the natural milieu of corneal stromal cells is fairly poor in nutrients, elevated glucose levels will predictably cause metabolic responses similar to those associated with hyperglycemia in obesity and diabetes, involving overproduction of reactive oxygen species that, when persistent, lead to mitochondrial fragmentation and, ultimately, apoptosis^34^. In the present study, cell death was also observed in corneal stromal cells undergoing scratch-wound repair in high-glucose, but not in low-glucose conditions. This effect was not immediate, starting in the restricted region of the original scratch three days after injury, and not affecting the remaining (distal) cells in culture. Interestingly, these phenomena closely resemble the intermediate-phase cell death process occurring in the stroma after corneal injury^35^. The somewhat fibroblastic appearance from cells in serum-free conditions at early stages might indicate that this effect was due to the release of growth factors and cellular debris from cells affected by the scratch procedure itself. Even considering that the dislodged cells are washed out after the scratch, it is possible that a few apoptotic/necrotic cells remained attached and ultimately released their contents into the supernatant. These contents include specific factors, such as IL1-alpha, known to be expressed by keratocytes in response to a wound, and capable of inducing these cells into a “fibroblastic repair phenotype”, and eventual apoptosis^35^,^36^. Although this process has been mostly attributed to cytokines, the precise mechanisms involved in keratocyte activation and intermediate-phase apoptosis remain unknown^36^. It is then feasible that, due to an incomplete restoration of the epithelial barrier function after injury *in vivo*, the concentration of glucose in the anterior stroma increases above normal physiological levels, i.e., via abnormal exposure to tear fluid. In cases where the loss of this barrier function persists, the resultant higher glucose availability could induce increased oxidative stress in cytokine-activated stromal cells, promoting cell death through mechanisms and with end-effects similar to those observed in our *in vitro* study. Overall, our results suggested that, in the future, it should be interesting to explore in detail the possible roles of high-glucose levels in keratocyte metabolism, as well as in corneal wound biology and regeneration.

Of additional import, this is, to our knowledge, the first report showing recovery of CD34 expression in corneal stromal cells cultured *in vitro*. CD34 has previously been described as an alternate keratocyte marker^37^ found to be down-regulated in injured corneas^38^ and irrevocably lost upon exposure to tissue culture conditions^21^. Although most commonly associated with haematopoietic stem cells^39^, CD34 is thought to be a key regulator of immune privilege, regulating cell proliferation, adhesion, and morphogenesis^40^. In the present study, LG conditions were shown not only to increase CD34 expression at both transcriptional and protein levels, but also to be instrumental to its post-translational modification. As evidenced by immunoblotting detection, the molecular weight of CD34 from cells in LG corresponded to the glycosylated form of the glycoprotein in the native tissue^37^, whereas in HG and BM conditions it corresponded to its protein core. This was relevant, as glycosylation has been shown to play a critical role on the specific functions of CD34^40^. In particular, CD34 was shown to contain a complex pattern of *O*-and *N*-glycans, including the sialyl-Lewis X epitope, a motif involved in E- and P-selectin binding and endothelial cell adhesion^41^. Interestingly, the glycosylation pattern of CD34 was shown to be variable and tissue-dependent^40^,^41^. As such, the use of low-glucose medium can also contribute to the study of CD34 glycosylation in the cornea and its particular but presently unknown function in keratocytes. The maintenance of CD34 expression has been previously demonstrated using media formulations such as M199 medium^42^. However, these effects were not directly attributed to the inherent low-glucose content of the formulation. Therefore, the results presented here make a considerable contribution to the interpretation of these previous studies.

Moreover, cells in LG showed increased expression of ALDH1A1 crystallin compared to HG conditions. This normally abundant corneal protein has been described as an essential component to match the refractive index of keratocytes to the ground substance of the corneal stroma, thus playing a critical role in the biological function of the organ^43^. Expression of corneal crystallins is thought to be essential in the resolution of corneal haze during embryonic development. At such time, the increased expression of corneal crystallins was shown to correspond with eyelid opening. Intriguingly, this developmental time-point also corresponds to a marked depression in the available metabolite concentration^44^,^45^. Therefore, the present data might be applied to the growing field of tissue engineering, specifically to the creation of bio-prosthetic corneal equivalents with higher crystallin levels for improved refractive properties and higher transparency.

Finally, this study presented evidences that the effects observed in cells grown in LG conditions were, at least partially, mediated by cAMP-dependent pathways. When supplementing HG medium with IBMX to increase intracellular cAMP levels, thus simulating LG conditions^46^, cells showed increased expression of CD34 and ALDH1A1. These results were in line with previous studies showing that CD34 and other keratocyte-characteristic markers respond to intracellular cAMP concentrations^9^,^11^,^23^,^47^. The cAMP-mediated transcriptional regulation has been shown to involve the interaction between cAMP response element binding proteins and cAMP response element (CRE) or CRE-like sequences within gene promoters^48^. However, in the conditions tested here, IBMX-induced elevated cAMP levels did not significantly alter the expression of keratocan or lumican, two keratocyte-characteristic markers with promoters containing CRE sequences. This might be explained by the complexity of glucose-related signalling cascades. Glucose has also been shown to regulate gene transcription directly through glucose response elements^49^ and indirectly through numerous other pathways^46^. Although preliminary, our results thus suggested that that the role of cAMP within keratocyte biology is more subtle, and that glucose affects the expression of the various keratocyte components through multiple pathways, with differing effects on cellular phenotype depending on the global signalling environment within the cell. As such, we defend the merit of future studies to elucidate in detail the molecular pathways regulating low-glucose effects in keratocytes.

## Conclusions

Overall, the present study showed that human corneal stromal cells were acutely sensitive to the extracellular concentration of glucose, and that low levels of this metabolite lead to the recovery of many phenotypic traits characteristic of keratocytes resident in healthy corneas. This study demonstrated the ability to revert the phenotype of corneal stromal cells, initially expanded in serum, from an activated fibroblastic form to a keratocyte-characteristic phenotype. This marked response to glucose concentration was in line with the hypothesis that the corneal stroma is particularly poor in nutrients, and that, by recapitulating this environment *in vitro*, it is possible to maintain cell viability for extended periods in culture. As such, we defend that low-glucose conditions constituted a significant improvement over the high-glucose serum-free media formulations used in this and other previous studies. These findings can also be directly applied to future investigations on the biology of the cornea, namely in studies assessing the role of chronically-low metabolite concentrations in keratocyte differentiation, stroma formation, and in the onset and progression of corneal diseases. In particular, studies of disease states such as keratoconus, in which cells are particularly difficult to maintain for extended periods *in vitro*, represent a promising target. Furthermore, dysregulation of nutrient mass transfer is often a symptom of anterior eye disorders such as Fuch’s dystrophy or epithelial injury. Such dysregulation may be subtle at first, and considered not clinically relevant. However, the perturbation of metabolite availability, especially in regards to increased glucose concentration of the stromal ground substance, may set up a system where the keratocytes of the corneal stroma are sensitised to injury, and compromise the immune privilege of the cornea, possibly through down-regulation of CD34 expression.

## Methods

### Isolation and initial expansion of human corneal stromal cells

Corneal stroma was excised from cadaverous human limbal tissue following removal of the central 7 mm for keratectomy (donors’ age between 31 and 89; average ± S.D. = 58 ± 15 year old). Corneal tissue was maintained at room temperature for 7-21 days before being debrided of both epithelium and endothelium, and then finely chopped using a scalpel. Corneal stromal cells were extracted by digesting the ECM components of the tissue in basal growth medium (BM) comprising DMEM:F12 (Life Technologies, CA, USA) with 5% foetal bovine serum (FBS; Biosera, FR), 1% penicillin/streptomycin (Life Technologies), 1 mM *L*-ascorbic acid 2-phosphate sesquimagnesium salt hydrate (Sigma-Aldrich, MO, USA), and initially supplemented with collagenase type-I (Life Technologies) at 2 g.L^−1^ (450 units.mL^−1^). The tissue was then incubated for 3 h on a rocker in a tissue culture incubator at 37 °C, 5% CO_2_, and 100% humidity. Stromal cells were then dissociated with 0.1% Trypsin-EDTA (Life Technologies) solution for 5 min and sieved through a 40 μm cell strainer (Thermo Scientific, UK). Finally, the Trypsin-EDTA solution was neutralised with BM, and cells pelleted at 500 × *g* for 5 min before being resuspended in BM, seeded into tissue culture flasks (Nunc; Thermo Scientific) and returned to the tissue culture incubator. Cultures had their medium replaced every two days until reaching 70% confluence, usually taking 4-5 days, upon which cells were dislodged using TrypLE Select (Life Technologies) and seeded for further expansion or serum-deprived to perform the various experiments. Corneal stromal cells were used up to passage 4.

### Media composition

The chemically-defined serum-free media (SFM) was prepared from DMEM low glucose (11880-028; Life Technologies). Briefly, DMEM containing 1 g.L^−1^ *D*-glucose was supplemented with 1 mM *L*-ascorbic acid 2-phosphate sesquimagnesium salt hydrate, 0.01 mg/ml human insulin, 55 μg/ml human transferrin, and 50 ng/ml sodium selenite (ITS; Sigma-Aldrich, MO, USA), 1% Glutamax (Life Technologies), and 1% penicillin/streptomycin, and subsequently adjusted to 2 or 4.5 g.L^−1^ of *D*-glucose (Sigma-Aldrich) to produce low-glucose (LG) or high-glucose, serum-free media (HG), respectively. LG medium was further supplemented with 2.5 g.L^−1^ of *D*-mannitol (Sigma-Aldrich), a sugar alcohol commonly used to replace glucose and maintain osmotic balance.

### Cell proliferation, morphology, and viability

To evaluate the effect of different glucose concentrations on corneal stromal cell proliferation and morphology, expanded cells were seeded into 24-well plates (Nunc) at a density of 5 × 10^4^ cells per well, maintained in either LG or HG for up to 21 days, with daily media change, and compared to cells cultured in BM. Cell proliferation was evaluated using the Alamar blue assay by incubating the different cultures with 0.5 mL of appropriate medium supplemented with 50 mM resazurin salt (R7017; Sigma-Aldrich). After 90 min incubation, 100 μl media aliquots were sampled in triplicate, and fluorescence read using a fluorometer (Fluostar, UK) with excitation at 530 nm and emission at 590 nm. The total number of cells was calculated from the linear correlation of a standard curve generated using 2 × 10^4^–1 × 10^6^ cells, and then normalised with the initial number of cells seeded. Cell viability was assessed at the end of the culture period using the Live/Dead cell double staining kit (Merck Millipore, DE), with live (Cyto-dye-positive) and dead (propidium iodide-positive) cells imaged in an Axiovert 200 microscope (Zeiss GMBH, DE). Cells were counted from ten fluorescence micrographs collected from random areas from each culture condition replicate. Cell morphology was evaluated throughout the period in culture by phase-contrast microscopy using a Nikon Eclipse inverted microscope (Nikon, JP) coupled with a Jenoptik CCD camera (Jenoptik AG, DE). All experiments were performed in triplicate, and using cells isolated from three independent donors.

### Cell migration

To evaluate the effect of different glucose concentrations on cell migration, corneal stromal cells were serum-deprived for three days prior to passage. Then, cells were dislodged as described above, suspended into LG, HG, or BM at 3 × 10^5^ cells.mL^−1^, and transferred in 0.5 mL aliquots into individual wells from 12-well plates (Nunc). Plates were kept in the tissue culture incubator overnight with an initial 45° tilt, to ensure that cells only adhered to the lower half surface of the wells. Afterwards, cultures were washed three times with sterile phosphate buffered saline (PBS) to remove any remaining unattached cells, and incubated for seven days with corresponding media. The cell-free surface in the immediate vicinity of cell-covered areas was imaged throughout the culture period using the LumaScope time-lapse inverted light microscope (Etaluma, CA, USA), and long-term cell migration quantified as surface area occupied by cells, normalised with corresponding total initial cell-free area. Area quantification was determined from binarised images using the ImageJ v1.7 software. Experiments were performed in triplicate, using cells isolated from three independent donors. Cell speed was evaluated by determining the position of 100 individual cells at 5 min intervals during the initial 24 h of migration, and tracing total distance covered by cells using the standard parameters of the wrMTrck plugin for ImageJ v1.3. Data was grouped in 10 μm.h^−1^ speed intervals, and expressed as the average of relative frequency from three independent experiments using cells from independent donors.

### Scratch-wound assay

Corneal stromal cells were seeded into 12-well plates at 3 × 10^5^ cells per well, attaching and forming a continuous monolayer after 4 h in culture. Cell monolayers were then serum-deprived for three days, washed with sterile PBS, and scratched using a 1 mL micropipette tip. Cell cultures were then washed thrice with PBS to remove floating cells and debris, and maintained in LG, HG, or BM for 7 days. The cell-free surface in the immediate vicinity of cell-covered areas was imaged throughout the culture period by phase-contrast microscopy using a Nikon Eclipse inverted microscope (Nikon), and wound closure quantified as the scratch area occupied by cells, normalised with corresponding total initial scratch area. Area quantification was determined from ten binarised images per condition using the ImageJ v1.7 software. Experiments were performed in triplicate, using cells isolated from three independent donors.

### Gel contraction assay

The contractile activity of corneal stromal cells was determined by evaluating contraction of cell-embedded collagen gels and comparing it with contraction of cell-free gels in the same conditions. Briefly, cells were serum-deprived for three days, dislodged with TrypLE, centrifuged and washed with sterile PBS to remove all traces of enzyme, and then embedded within uncompressed or compressed collagen gels, as previously described^50^,^51^. Briefly, a solution composed of ice-cold rat tail collagen type-I (2 g.L^−1^ in 0.6% acetic acid; First Link Ltd, UK) mixed with 10 × Modified Essential Medium (Life Technologies, USA) and neutralised with 1 M NaOH at a 1:7:1:1 volume ratio was used to resuspend a cell pellet (1 × 10^5^ cells, passage 3–4, per mL^−1^ of gel solution), and then poured into 12-well cell culture plates (2 mL per well) and allowed to polymerise for 30 minutes at 37 °C and 5% CO_2_. This cellular density enabled cell-cell interactions without overcrowding. Plastic compression was performed after polymerisation by placing individual gels between two layers of gauze and over a 1 mm-thick metal grid topping 10 plus sheets of absorbent filter paper, and then applying a 134 g load for 5 min at room temperature. Both compressed and uncompressed collagen gels containing cells were then transferred to 6-well plates and incubated in LG, HG, or BM conditions for 15 days, with daily replacement of media, and imaged at the beginning and end of the culture period to evaluate gross changes in gel size. Gels prepared by a similar process but without cells were used to exclude the contribution of culture media to gel contraction. Area determination was performed from binarised images using the ImageJ v1.7 software. Experiments were performed in duplicate, using cells isolated from three independent donors.

### RNA extraction and qPCR

The molecular phenotype of corneal stromal cells maintained in LG, HG, and BM was analysed by qPCR due to the low abundance of protein markers at the early stages of culture. Total RNA from cells grown in LG, HG, or BM for 9 days was extracted with the RNEasy mini kit (Qiagen, NE), as per manufacturer’s instructions. RNA concentration was determined with a Nanodrop 2000 UV/Vis spectrometer (Thermo Scientific) and 1 μg of RNA was transcribed to cDNA using the Verso cDNA synthesis kit (Thermo Scientific) in a total reaction volume of 20 μl. Quantitative real-time PCR (qPCR) was carried out with SYBR green-based QuantiTect SYBR Green PCR Kit mix (Qiagen). Specific primer pairs were designed and validated in terms of efficiency (Table 1) by single-peak melting curves and fragment size analysis using an Eco thermo cycler (Illumina, CA, USA), with a three-step PCR reaction with melt curve. Relative expression was determined using the Eco-study programme (Illumina) with a modified Pfaffel equation to include primer efficiency^52^. Expression values were relative to the housekeeping gene *POLR2B* (NCBI Entrez reference 5431). Experiments were performed in duplicate using cells isolated from five independent donors, passages 2–4.

### Protein extraction and immunoblotting analysis

Cells maintained in LG, HG, or BM for 21 days were lysed in RIPA buffer (25 mM Tris-HCl pH 7.6, 150 mM NaCl, 1% NP40, 1% sodium deoxycholate, 0.1% SDS; Sigma-Aldrich) supplemented with 1 × Complete Protease Inhibitor Cocktail (Roche, CH) for 30 min on ice. Lysate from corneal stroma was obtained by dissociating central corneal stromal tissue in RIPA buffer using the gentleMACS Dissociator (Miltenyi Biotec, DE) for 1 min, followed by additional 30 min incubation on ice. Protein concentration was determined using the BCA protein assay (Bio-Rad, CA, USA). Cell lysates were loaded into 10% Criterion XT Pre-Cast gels (Bio-Rad) (10 μg total protein per well) and run by reducing SDS-PAGE. Proteins were transferred to PVDF membranes (Thermo Scientific) using a wet Western blotting system (Bio-Rad) and blocked for 1 h in Tris-buffered saline containing 0.1% Tween 20 (TBS-T) and 5% bovine serum albumin (BSA; First Link, UK). Membranes were incubated overnight with 1:500 rabbit anti-CD34 (ab81289; Abcam, UK), anti-keratocan (sc66941; Santa Cruz Biotechnology, CA, USA), 1:1000 mouse anti-αSMA (VP-S281; Vector Labs, UK) or anti-α-tubulin (T6793; Sigma-Aldrich) primary antibodies in blocking buffer, washed 4 × 15 min in TBS-T, and then correspondingly incubated for 2 h at room temperature with HRP-coupled anti-rabbit or anti-mouse secondary antibodies (R&D) diluted 1:2000 in blocking buffer. After a second 4 × 15 min wash series, membranes were incubated 5 min with Immobilon Western Chemiluminescent HRP Substrate (Merck Millipore) and imaged using the ImageQuant LAS 4000 mini system (GE Healthcare, UK). Protein expression was determined from non-saturated images by densitometry analysis using the ImageJ v1.7 software, and normalised relative to expression of α-tubulin loading control. Experiments were performed in duplicate, using cells isolated from three independent donors.

### Immunofluorescence microscopy

Corneal stromal cells seeded onto glass coverslips were grown as described above in LG, HG, or BM, washed twice in PBS, and fixed in 4% paraformaldehyde in PBS for 20 min at room temperature. Cells were then permeabilised in PBS containing 0.1% Triton X-100 (PBS-T) for 5 min, blocked with PBS containing 2.5% goat serum and 2.5% bovine serum albumin for 1 h, incubated overnight at 4 °C with rabbit anti-ALDH1A1 monoclonal antibody (ab52492; Abcam) diluted 1:1000 in blocking buffer, washed again for 3 × 15 min in blocking buffer, and incubated with the Alexa 488-conjugated goat anti-rabbit IgG antibody (Life Technologies) diluted 1:1000 in blocking buffer for 2 h at room temperature. The secondary antibody was washed for 15 min in PBS before adding Alexa 594-conjugated phalloidin (Life Technologies) diluted 1:400 in blocking buffer. Cells were then washed for 2 × 15 min before being mounted on slides with DAPI-containing mounting media (Vector Labs). Negative-IgG (rabbit monoclonal anti-NF-M; ab92539; Abcam) and no-primary antibody controls were performed in parallel (Supplementary Fig. S4). Cells were visualised on a Zeiss Axio imager A1 (Zeiss) with a × 20 objective, with images collected on the Axiovision v.4.8 image analysis software. Experiments were performed in duplicate, using cells isolated from five independent donors.

### Phosphodiesterase inhibition assay

Corneal stromal cells seeded in multiwall dishes or on glass coverslips were maintained in BM for two days, washed twice with PBS, and then grown for two additional days in HG supplemented with 5 × 10^−6^ M of phosphodiesterase inhibitor 3-isobutyl-1-methylxanthine (IBMX; Tocris, UK) to elicit increase of the intracellular levels of cyclic adenosine monophosphate (cAMP). The corresponding volume of dimethyl sulphoxide (DMSO) was used as a vehicle control. Cells in LG or BM were used as positive and negative controls, respectively. After incubation, cell cultures were washed twice in PBS and analysed for cell morphology by phase-contrast microscopy, gene transcription by qPCR, and protein expression by immunofluorescence microscopy, as described above. Experiments were performed in duplicate, using cells isolated from three independent donors.

### Statistical analysis

Error bars represent the standard deviation of the mean, analysed *a priori* for homogeneity of variance. Replicates from each independent experiment were confirmed to follow a Gaussian distribution, and differences between groups were determined using one-way analysis of variance (ANOVA) followed by Bonferroni’s multiple comparison *post hoc* tests. Significance between groups was established for *p* < 0.05, 0.01, and 0.001, with a 95% confidence interval, and corresponding R^2^ values (percentage of variation explained by medium condition) of 0.92 and 0.98 (cell proliferation, day 14 and 21), 0.92 (viability), 0.90 and 0.99 (migration, day 3 and 7), 0.97 and 0.93 (wound-closure, day 3 and 7), 0.99 (contraction), and 0.79–0.98 and 0.93–0.97 (gene and protein expression assays, respectively).

## Additional Information

**How to cite this article**: Foster, J. W. *et al*. Low-glucose enhances keratocyte-characteristic phenotype from corneal stromal cells in serum-free conditions. *Sci. Rep*. **5**, 10839; doi: 10.1038/srep10839 (2015).

## Supplementary Material

Supplementary Information

## Figures and Tables

**Table 1:** Gene markers and corresponding primers.

Gene	Accession No.	Forward sequence (*3*’-*5*’)	Reverse sequence (*3*’-*5*’)
*KERA*	NM_007035	TATTCCTGGAAGGCAAGGTG	ACCTGCCTCACACTTCTAGACC
*LUM*	NM_002345.3	CCTGGTTGAGCTGGATCTGT	TAGGATAATGGCCCCAGGA
*COL5A1*	NM_000093.3	ATCTTCCAAAGGCCCGGATG	AAATGCAGACGCAGGGTACA
*CD34*	NM_001025109.1	CTTGGGCATCACTGGCTATT	TCCACCGTTTTCCGTGTAAT
*ALDH1A1*	NM_000689.4	CTCTCACTGCTCTCCACGTG	GAGAAGAAATGGCTGCCCCT
*ALDH3A1*	NM_001135168	CCCCTTCAACCTCACCATCC	GTTCTCACTCAGCTCCGAGG
*POLR2B*	NM_000937.4	CATCATCCGAGACAATGGTG	AACAATGTCCCCATCACACA
