# Supplementary material for: Low-glucose enhances keratocyte-characteristic phenotype from corneal stromal cells in serum-free conditions
Source: Sci Rep. 2015 Jun 3;5:10839. doi: 10.1038/srep10839 (PMC4650697; doi:10.1038/srep10839)
Supplement: Supplementary Information [file srep10839-s1.doc]

**Low-glucose enhances keratocyte-characteristic phenotype from corneal stromal cells in serum-free conditions**

James W. Foster, Ricardo M. Gouveia, Che J. Connon

**Supplementary Figures**


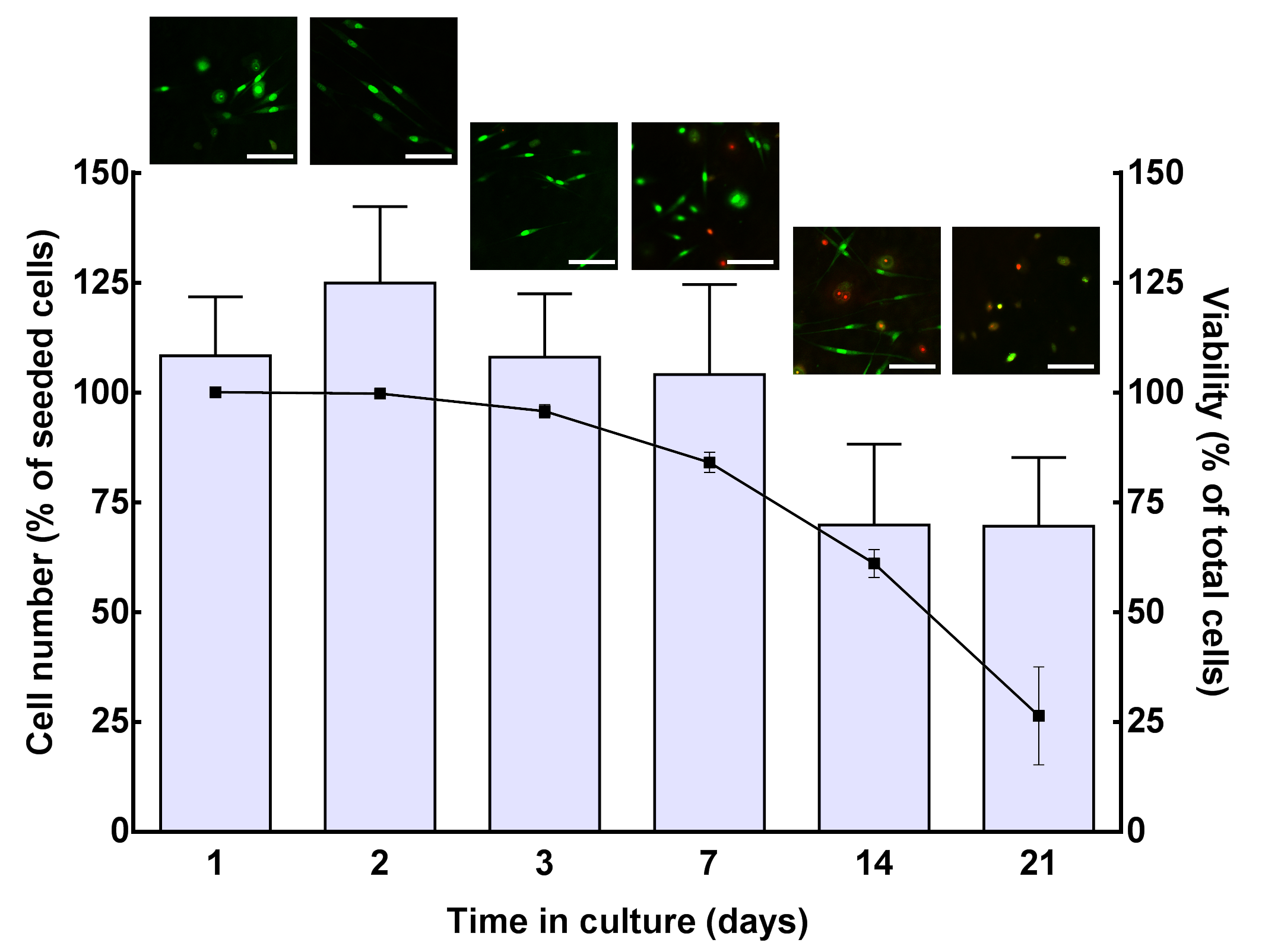


**Figure S1:** *Effects of 1 g.L^-1^ of glucose on human corneal stromal cell proliferation and viability.* Human corneal stromal cells were grown in serum-free media containing 1 g.L^-1^ of *D*-glucose for up to 7 days. Cell number (*bars*, *left* y*-axis*) was determined using the Alamar blue assay, normalised against the number of cells initially seeded. Cell viability was analysed using the live/dead double staining assay and quantified from fluorescence micrographs as percentage of live cells over total number of cells (*line, right* y-*axis*). Representative images of live (*green*)/dead cell (red) staining are shown for each time point. Data was expressed as average ± S.D. of three independent experiments (*n* = 3). Scale bars = 100 µm.


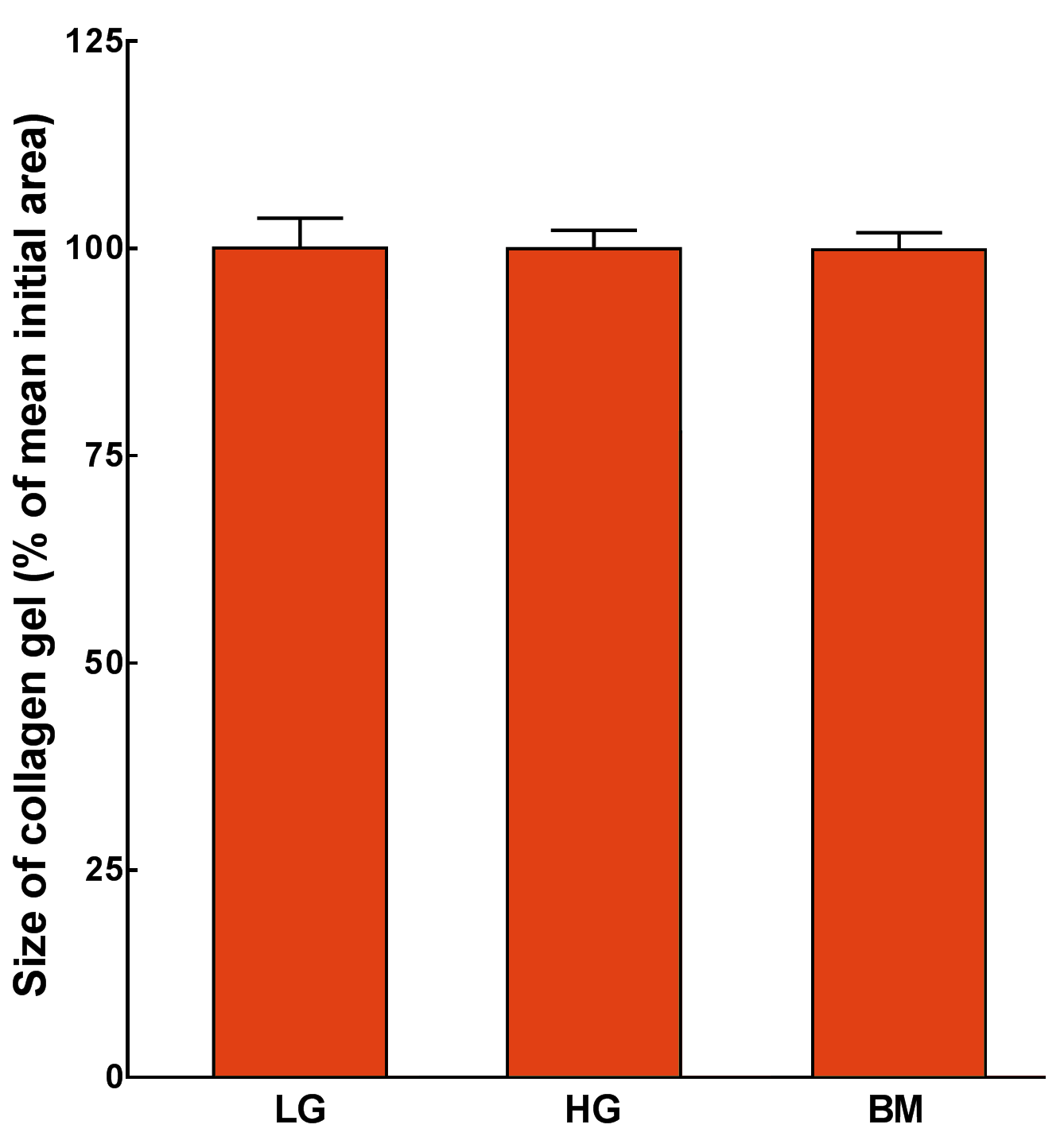


**Figure S2:** *Effect of glucose concentration on the contractile activity of compressed collagen gels.* Collagen gels without cells were compressed and then maintained in LG, HG, or BM conditions for 15 days. Contraction was quantified as variation between initial and final size of collagen gels. No variation in collagen gel size due to different media was observed. Data was expressed as average ± S.D. of duplicates from three independent experiments (*n* = 3); *** corresponded to *p*<0.001.


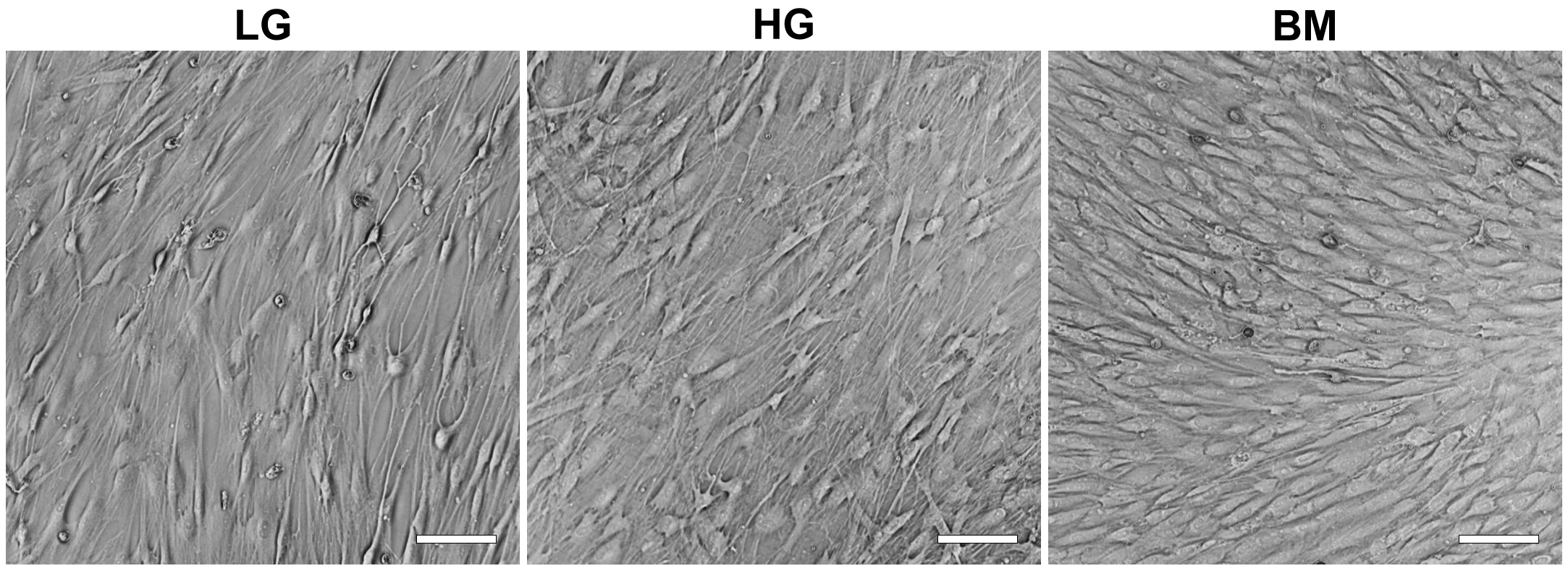


**Figure S3:** *Effect of glucose concentration on scratch-wound closure*

Serum-deprived human corneal stromal cells seeded as a monolayer were scratched and then maintained in LG, HG, or BM for 7 days. Representative micrographs of areas not affected by the scratch were obtained in triplicate from three independent experiments, and showed healthy confluent cultures, with cell morphologies comparable to those observed in the growth curve assays (Fig. 1). Scale bars = 100 µm.


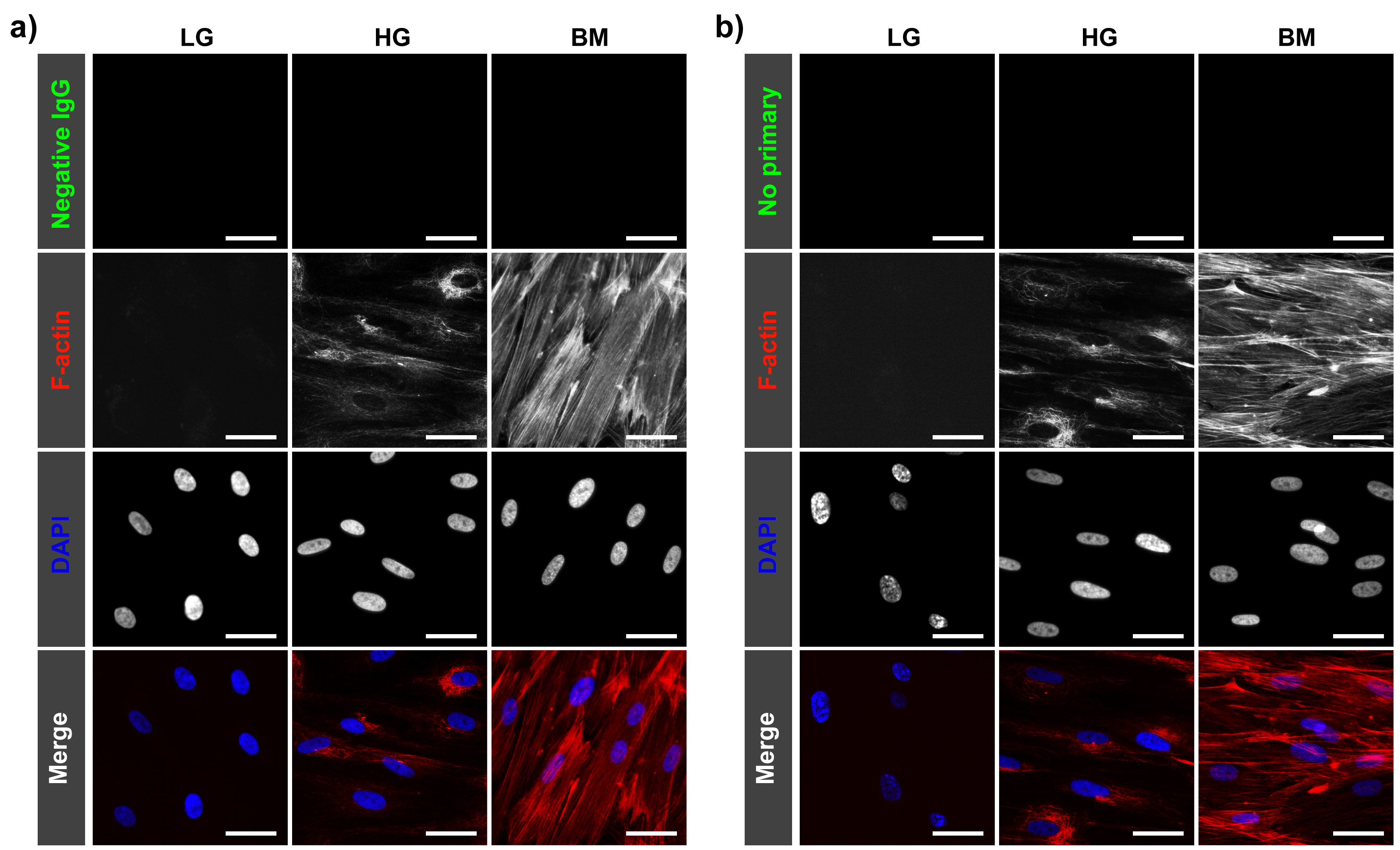


**Figure 6:** *Fluorescence microscopy analysis of human corneal stromal cells grown in LG, HG, or BM.*

Human corneal stromal cells grown in LG, HG, or BM were analysed by fluorescence microscopy using a) negative-IgG (anti-NF-M) and b) no-primary antibody controls (*green*), along with F-actin (*red*) and nuclei staining (*blue*). The absence of signals from the green channel indicated that green fluorescence reported in Fig. 6 was specific for ALDH1A1 and not the result of unspecific antibody binding, auto-fluorescence, or spectral bleed-through. Panels show representative images of three independent experiments. Scale bars = 20 µm.
